# Effect of Weekend Admissions on the Treatment Process and Outcomes of Internal Medicine Patients: A Nationwide Cross-Sectional Study

**DOI:** 10.1097/MD.0000000000002643

**Published:** 2016-02-12

**Authors:** Chun-Che Huang, Yu-Tung Huang, Nin-Chieh Hsu, Jin-Shing Chen, Chong-Jen Yu

**Affiliations:** From the Institute of Health Policy and Management, College of Public Health, National Taiwan University, Taipei (C-CH); Master Degree Program in Aging and Long-Term Care, Kaohsiung Medical University, Kaohsiung (Y-TH); Department of Internal Medicine (N-CH, C-JY); Division of Hospital Medicine (N-CH, J-SC); Department of Traumatology; and Department of Surgery, National Taiwan University Hospital (J-SC), Taipei, Taiwan.

## Abstract

Many studies address the effect of weekend admission on patient outcomes. This population-based study aimed to evaluate the relationship between weekend admission and the treatment process and outcomes of general internal medicine patients in Taiwan.

A total of 82,340 patients (16,657 weekend and 65,683 weekday admissions) aged ≥20 years and admitted to the internal medicine departments of 17 medical centers between 2007 and 2009 were identified from the Taiwan National Health Insurance Research Database. A generalized estimating equation (GEE) analysis was used to compare patients admitted on weekends and those admitted on weekdays.

Patients who were admitted on weekends were more likely to undergo intubation (odds ratio [OR]: 1.27; 95% confidence interval [CI]: 1.16–1.39; *P* < 0.001) and/or mechanical ventilation (OR, 1.25; 95% CI, 1.15–1.35; *P* < 0.001), cardio-pulmonary resuscitation (OR: 1.45; 95% CI: 1.05–2.01; *P* = 0.026), and be transferred to the intensive care unit (ICU) (OR: 1.16; 95% CI: 1.03–1.30; *P* = 0.015) compared with those admitted on weekdays. Weekend-admitted patients also had higher odds of in-hospital mortality (OR: 1.19; 95% CI: 1.09–1.30; *P* < 0.001) and hospital treatment cost (OR: 1.04; 95% CI: 1.01–1.06; *P* = 0.008) than weekday-admitted patients.

General internal medicine patients who were admitted on weekends experienced more intensive care procedures and higher ICU admission, in-hospital mortality, and treatment cost. Intensive care utilization may serve as early indicator of poorer outcomes and a potential entry point to offer preventive intervention before proceeding to intensive treatment.

## INTRODUCTION

Weekend admissions are associated with higher risk of mortality in patients admitted to the intensive care unit (ICU)^1–4^ and the internal medicine unit.^5^ Most patients admitted to the Internal Medicine Department are elderly and have multiple comorbidities.^6^ The status of some patients may worsen, resulting in a need for endotracheal intubation, invasive mechanical ventilation, and/or invasive monitoring during hospitalization.^7^ Critically-ill patients may experience a spectrum of different life-threatening conditions that require unplanned ICU transfers, tracheostomies, or cardio-pulmonary resuscitation (CPR).^8,9^ Although treatment delays have been observed in patients admitted on weekends for a variety of diseases,^10,11^ knowledge of the incidence of intubation and other intensive interventions in internal medicine patients admitted on weekends is important.

However, few studies have specifically explored the relationship between weekend admission and treatment interventions during hospitalization for general internal medicine patients. When assessing hospitalization outcomes, most studies also adjusted for patient characteristics but not for the hospital accreditation level.^7^ Choosing hospitals with similar level, facility, and staffing, such as studies done for stroke centers,^12^ should be considered. To investigate the so-called weekend effect on intensive treatment utilization, this study used a nationwide population-based sample to examine the relationship between weekend admission and the use of intubation and other intensive interventions in patients who were admitted to the internal medicine departments of medical centers in Taiwan.

## MATERIALS AND METHODS

### Data Source and Study Population

This retrospective population-based cross-sectional study retrieved data from the National Health Insurance Research Database (NHIRD) in Taiwan covering the period between 2007 and 2009. The data included in-patient expenditures by admission and the registry for contracted medical facilities. Links of the datasets to relevant variables were preserved through scrambled unique personal or hospital identification numbers. All individually identifiable health information was encrypted by the National Health Insurance Administration (NHIA) to protect privacy and assure confidentiality. The procedural and diagnostic codes were classified according to the International Classification of Diseases, Ninth Revision, Clinical Modification (ICD-9-CM) coding system. The NHIA outlined the requirements for the standardized procedures and experts performed a quarterly review to ensure quality of care and accuracy of the claim files. The Research Ethics Committee of National Taiwan University Hospital approved this study (Approval No. 201307015W, Taiwan).

**FIGURE 1:**
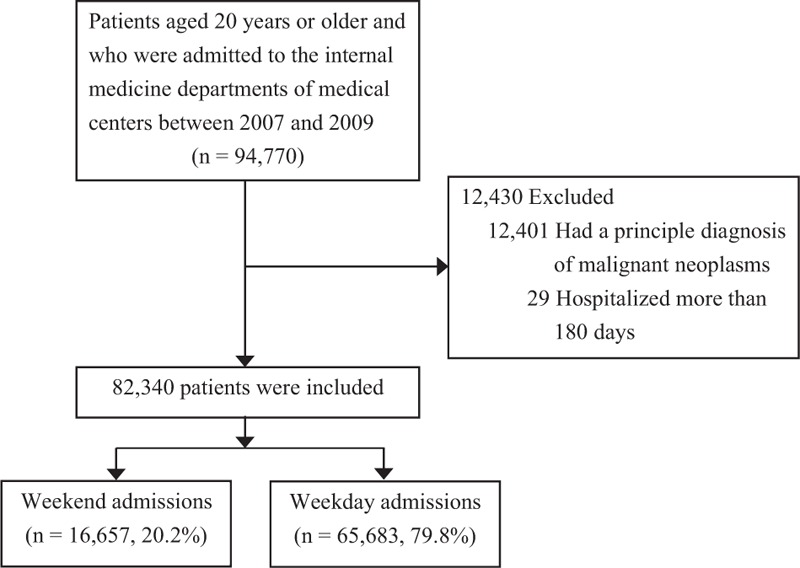
The selection of study patients. Patients who were admitted to the internal medicine departments of medical centers on weekdays and weekends were identified.

A total of 94,770 patients aged ≥20 years or older and were admitted to the internal medicine departments of medical centers in Taiwan between 2007 and 2009 were identified. After excluding 12,401 (13.1%) patients with a principal diagnosis of malignant neoplasms (ICD-9-CM codes, 140–239) because their in-hospital mortality could be affected by malignancy and 29 (<0.01%) patients who were hospitalized for >180 days in order to reduce outlier effects, the final sample had 16,657 patients admitted on weekends and 65,683 admitted on weekdays (Figure 1).

### Study Outcomes and Covariate Measurements

The primary endpoint was the use of general intensive care interventions, including intubation (ICD-9-CM procedure code, 96.04 and 96.05), mechanical ventilation (96.7), CPR (99.60 and 99.63), and ICU transfer during the hospitalization.^13^ These ICD-9-CM procedure codes were reliable and essential for reimbursement and were verified by the NHIA. The secondary endpoints were the length of the hospital stay, in-hospital mortality, and hospital treatment cost.

The independent variable was the patient's day of admission, which was used to compare patients with weekend and weekday admissions. A weekend admission was defined as an admission to the Internal Medicine Department between 12:01 am Saturday through 11:59 pm on Sunday.^14^ All of the other admissions were considered weekday admissions.

Covariates selected according to literature review were patient characteristics (eg, age, sex, low-income family status based on the patient's eligibility for Taiwan's National Health Insurance premium for low-income households, principal diagnoses, co-morbidities, and year of admission) and the characteristics of medical centers (ie, ownership, patient volume, and geographic location).

*The principal* diagnoses *were classified into several disease categories: pneumonia (ICD-9-CM codes 480–486 or 507), urinary tract infection (UTI, 590.1, 595.0, 595.9, or 599.0), ischemic heart disease (IHD, 410–414), congestive heart failure (CHF, 428), upper gastrointestinal bleeding (530–535 or 578), acute exacerbation of chronic obstructive pulmonary disease (COPD, 490–492 or 496), stroke (430–438), cellulitis (681 and 682), and other* diseases.^12^*Disease severity was associated with increased risk of ICU transfer, prolonged LOS, and death. The* modified Charlson co-morbidity index (CCI) was used to adjust the case mix for the severity of co-morbid illness with administrative data,^15^ a method widely applied by other researchers.^1,5,12^

The registry for the contracted medical facilities provided information about the ownership and location of the medical centers. All of the medical centers in Taiwan were public or not-for-profit hospitals. Their locations were classified into 4 regions (northern, central, southern, and eastern) according to the National Statistics of Regional Standard Classification. Annual patient volume was defined as the average number of internal medicine patients treated by the medical centers during the 3-year study period. Using a cutoff point at the 60th percentile of volume annually, *medical centers that treated an average of <1800 and ≥1800* internal medicine *patients annually were classified as low- and high-volume medical centers, respectively.*

### Statistical Analysis

All statistical analyses were conducted using the SAS version 9.3 (SAS Institute, Cary, NC). Use of intubation and other intervention and hospitalization outcomes, and patient demographics and institutional characteristics between weekend and weekday admissions were examined. A generalized estimating equation (GEE) with an exchangeable correlation structure was used to account for repeated measurements on the same medical center. The GEE analysis estimated adjusted odds ratios (OR) with 95% confidence intervals (CI) of the status of weekend admissions for the outcome variables. Statistical significance was set at *P* *<* 0.05.

## RESULTS

### Patient Demographics and Hospital Characteristics

There were 82,340 patients who were admitted to the Internal Medicine Departments of 17 medical centers in Taiwan, including 16,657 (20.2%) weekend admissions and 65,683 (79.8%) weekday admissions. Comparisons of the patient demographics, clinical factors, and institutional characteristics between the weekday and weekend admissions (Table 1) revealed a greater proportion of weekend admissions were >65 years of age (57.1%) compared to weekday admissions (55.4%) (*P* < 0.001). Compared with weekday admissions, weekend admissions also had higher percentages of patients who were diagnosed with pneumonia, urinary tract infection, and/or cellulitis. The percentage of patients with low Charlson Co-morbidity index (CCI) score (≤2) was higher among those who were admitted on weekends (87.4%) than among those admitted on weekdays (85.8%) (*P* < 0.001).

**TABLE 1:**
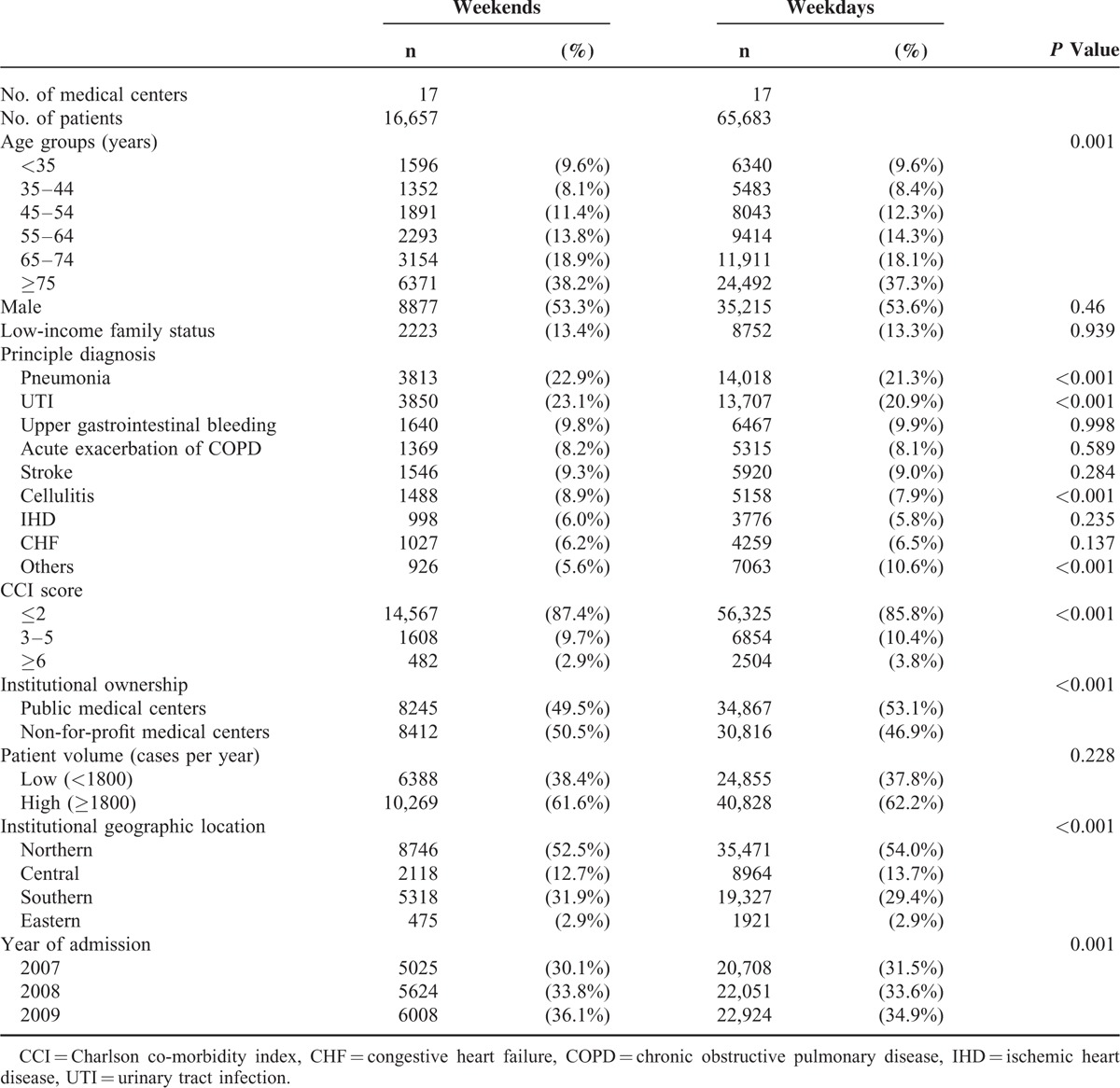
The Patient and Institution Characteristics of Patients Admitted on Weekends and Weekdays

In terms of institutional characteristics, patients who were admitted on weekends were more likely to be treated at medical centers that were not-for-profit and that had low patient volume (Table 1).

### Treatment Processes and Outcomes

General internal medicine patients who were admitted on weekends had a higher percentage of receiving intubation, mechanical ventilation, CPR, and ICU transfer. In-hospital mortality and hospital treatment costs of weekend admissions were higher than those of weekday admissions (Table 2). After adjusting for patient demographics, clinical factors, and institutional characteristics, multivariate GEE analyses revealed that internal medicine patients who were admitted on weekends were more likely to undergo intubation (OR: 1.27; 95% confidence interval [CI]: 1.16–1.39; *P* < 0.001), mechanical ventilation (OR: 1.25; 95% CI: 1.15–1.35; *P* < 0.001), CPR (OR: 1.45; 95% CI: 1.05–2.01; *P* = 0.026), and/or transfer to the ICU (OR: 1.16; 95% CI: 1.03–1.30; *P* = 0.015) during their hospitalization than those who were admitted on weekdays (Table 2).

**TABLE 2:**
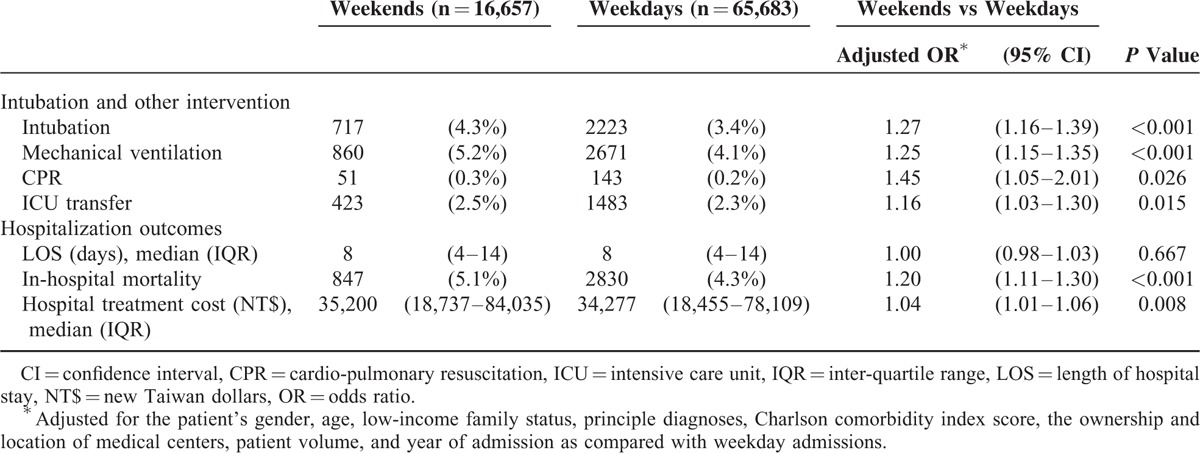
Incidence of Intubation and Other Interventions and Hospitalization Outcomes Between the Patients With Weekend and Weekday Admissions

Furthermore, after covariate adjustment, the weekend admissions had significantly increased odds of in-hospital mortality (OR: 1.20; 95% CI: 1.11–1.30; *P* < 0.001) and hospital treatment cost (OR: 1.04; 95% CI: 1.01–1.06; *P* = 0.008) compared to weekday admissions (Table 2).

## DISCUSSION

Internal medicine patients who were admitted on weekends were significantly more likely to undergo intubation, mechanical ventilation, and CPR, and to be transferred to the ICU during hospitalization compared with those who were admitted on weekdays, even after adjusting for the relevant covariates. Moreover, after covariate adjustment, weekend admissions had increased odds of in-hospital mortality and hospital treatment cost. To date, this is the first study to use a nationwide representative sample to assess the utilization of intubation and other intensive care interventions among general internal medicine patients admitted on weekends at medical centers throughout Taiwan. These intensive care interventions are common for general medicine patients who have deteriorating clinical conditions during hospitalization.

The adverse outcomes of weekend admissions may be due to the decreased staffing at medical centers on weekends compared to weekdays.^1,7,10^ However, a decreased staffing level is only one of the factors that potentially explain the poor outcomes in patients admitted on weekends.^16^ Prior studies have reported that the adverse outcomes of weekend admissions may be due to delayed invasive diagnostic and therapeutic procedures, limited access to primary intensive care measures,^10,17^ and selection biases that stem from a general reluctance of patients to solicit care during the weekend.^7^ A general impression is that the therapeutic procedures and intensive care may be limited on weekends, causing an inferior quality of care.

Consistent with findings of previous studies, this study shows that internal medicine patients admitted on weekends have a higher in-hospital mortality.^1,5^ The increased incidences of intubation, mechanical ventilation, CPR, and ICU transfer may actually serve as preludes to in-hospital mortality. These interventions are usually predictors of prognosis in patients with severe acute conditions and can serve as more sensitive and practical quality indicators of hospitalization. Therefore, improving the early detection of unstable conditions in hospitalized patients through techniques such as a clinical warning system^18,19^ or a rapid response team^20,21^ may curb the deteriorating course and prevent ICU transfer and eventual death.

Nonetheless, this study has several limitations. First, it was unable to identify the precise timing of the intubation and other intensive interventions that the internal medicine patients received during their hospitalization. It is possible that internal medicine patients are more likely to receive intensive treatments on weekdays than on weekends, regardless of admission. Second, intubation and other intensive interventions that were identified by the ICD-9-CM procedural codes in the administrative data may be found less frequently in patients admitted to internal medicine departments. The low incidence would result in an underestimation of the statistical significance of the differences between the patients with weekday and weekend admissions. Thus, the true differences between weekend and weekday admissions may be even larger than those that observed in this study. Third, information on clinical assessment of disease severity was not available and severity was derived from calculation of a CCI score. This might have had a direct effect on patient outcomes. Lastly, the therapeutic patterns vary significantly across institutions.^22^ In this study, enrollment of only medical centers in a nationwide sample cannot completely control this variation.

In conclusion, this study provides evidence that internal medicine patients admitted on weekends experience increased risks of treatment with endotracheal intubation, mechanical ventilation, CPR, ICU transfer, in-hospital mortality, and higher hospital treatment cost compared with those admitted on weekdays. These findings suggest that efforts to recognize high-risk weekend-admitted patients and prevent life-threatening events in weekend admissions are imperative. The use of intubation and other intensive interventions during hospitalization may indicate the need for earlier intervention before any kind of deterioration.
